# A Case of Mucinous Cystadenofibroma of the Ovary

**DOI:** 10.1155/2014/130530

**Published:** 2014-03-17

**Authors:** Dae Hyung Lee

**Affiliations:** Department of Obstetrics and Gynecology, Yeungnam University School of Medicine, Daegu, Republic of Korea

## Abstract

Ovarian adenofibroma is a rare benign tumour originating from the germinal lining and stroma of the ovary. We describe here the case of a 36-year-old woman with an ovarian mucinous cystadenofibroma that was diagnosed as a benign cystic mass of the ovary before surgery. The tumour was a cystic lesion composed of 2 regions: one filled with mucinous fluid and the other with yellowish solid components. The patient successfully underwent a left oophorectomy.

## 1. Introduction

Ovarian cystadenofibroma is a very rare benign tumour that originates in the epithelium and includes diverse structures composed of cystic and solid fibrotic tissues. These tumours are classified, according to the epithelial cell types present, as serous, endometrioid, mucinous, clear cell, and mixed categories. Because ovarian cystadenofibroma presents as a multicystic mass with solid components, preoperative differential diagnosis is important to distinguish it from malignant neoplasms. In this report, we describe the treatment of a 36-year-old woman with an ovarian cyst. The lesion was not diagnosed as a benign tumour before surgery. After surgery, the tumour was diagnosed as a mucinous cystadenofibroma.

## 2. Case

A 35-year-old gravid (1-0-0-1) woman presented with left lower abdominal pain for several years. Her menstrual cycle length was 30 days, menstruation volume was normal, and she experienced slight menstrual pain. Her family history was uneventful. Upon comprehensive medical testing, an ovarian mass was detected by pelvic CT. After the patient was transferred to our hospital, she underwent pelvic MRI. The MRI showed a 6 cm left ovary mass with numerous small cysts and a fibrous septal structure.

At the time of admission, the patient's blood pressure was 140/80 mmHg, her pulse was 74 bpm, her temperature was 36.5°C, and her general condition was good. During a pelvic examination, a painless cystic mass was detected in the left adnexal area. Blood test results were normal: haemoglobin level, 12.9 g/dL; red blood cell volume, 38.7%; leukocyte count, 6.380; and platelet count, 235,000. Results of urinalysis and blood chemistry analysis were within normal ranges. Serum concentrations of the tumour markers CA-125 and CA19-9 were 18.08 and 55.72 ng/mL, respectively; the CA19-9 tumour marker level was slightly elevated compared to normal. Transvaginal sonography performed at our hospital showed that the uterus was deviated to the right; no other notable features were observed, except for the left ovarian tumour, approximately 6 × 6 cm in size, with a septum. The inside of the tumour displayed irregular contrast. Approximately two-thirds of the tumour was cystic, and partially solid patterns were detected. The right adnexa could not be observed due to the left ovarian tumour.

**Figure 1:**
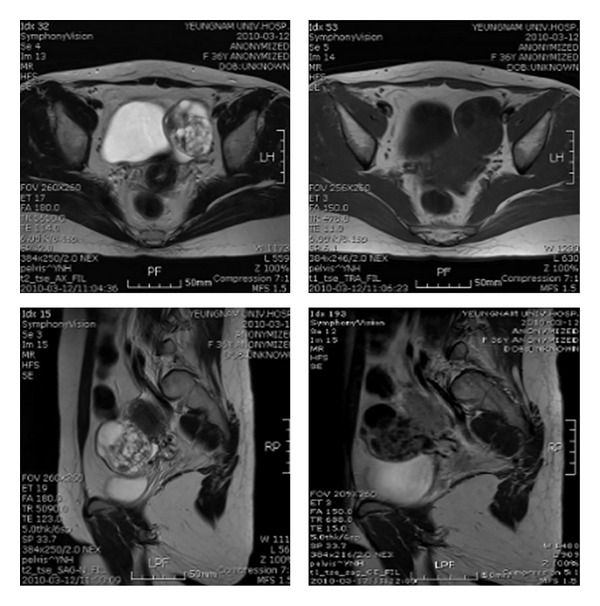
Pelvic MRI before surgery. Image showed approximately 6 cm diameter mass with numerous small cysts and fibrous septal structure in the left ovary.

A pelvic MRI showed a cystic tumour, approximately 6 cm in diameter with numerous small cysts and a fibrous septal structure. Contrast-enhanced images revealed prominent features of the wall and the septum of the mass. Mild and diffuse thickening of the junctional zone was observed (Figure 1).

The patient underwent laparotomy surgery with a low midline incision. The uterus was full-term sized, with a smooth surface. A whitish cyst approximately goose egg size was observed in the left ovary. The mass was very hard and the surface was relatively smooth. Biopsy specimens were examined as frozen sections, leading to the diagnosis of mucinous cystadenofibroma.

**Figure 2:**
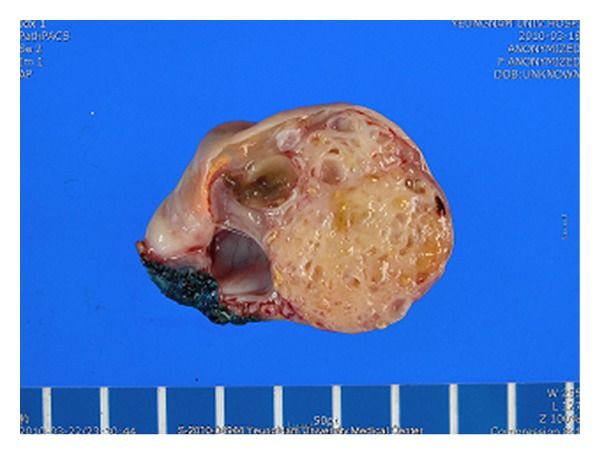
Photograph of the left ovarian tumour and the cut surface. Multiloculated cystic tumour with additional solid areas containing small cysts.

**Figure 3:**
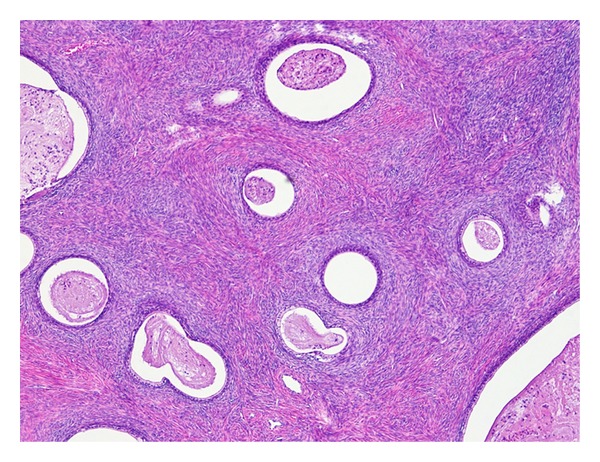
Relatively uniform mucinous glands are associated with a prominent fibrous stroma (×40, H&E stain).

**Figure 4:**
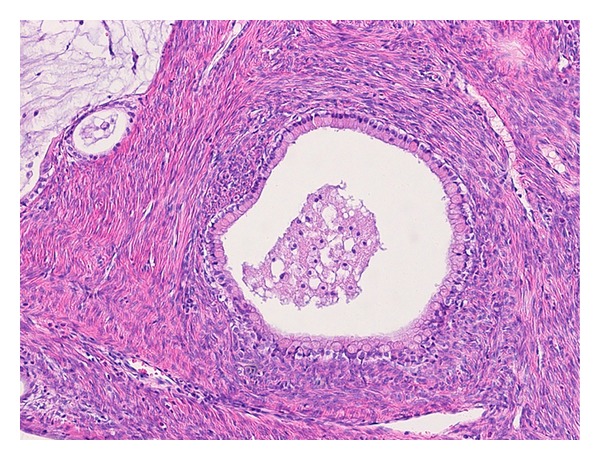
The tumour is composed of glandular and cystic portions lined by mucinous columnar epithelium, with mucinous fluid in the lumen and a predominantly fibromatous stroma (×100, H&E stain).

A view of the bisected surface showed a multiloculated cystic tumour with more solid areas containing small cysts (Figure 2). Relatively uniform mucinous glands were associated with a prominent fibrous stroma (Figure 3). The tumour was composed of glandular and cystic portions lined by mucinous columnar epithelium, with mucinous fluid in the lumen and a predominantly fibromatous stroma (Figure 4).

After surgery, the patient was discharged without specific symptoms. The patient did not receive any additional treatment beyond regular checkups.

## 3. Discussion

Although ovarian cystadenoma is a common benign tumour, ovarian cystadenofibroma is relatively rare. The actual incidence of ovarian cystadenofibromas is unknown. Primary ovarian cystadenofibromas are encountered in women aged between 15 and 65 years [1]. Cho et al. estimated that these tumours account for approximately 1.7% of all benign ovarian neoplasms [2].

Ovarian cystadenofibroma is a type of surface epithelial tumour. These tumours exhibit a fibrous stroma in variable amounts in all subtypes [3]. When the stroma occupies an area greater than the cystic portion, the suffix “-fibroma” is added, as in “serous adenofibroma.” The presence of more than 1 cyst over 1 cm in diameter warrants use of the prefix “cyst” as in “cystadenofibroma.” These tumours are classified, according to the epithelial cell types present, as serous, endometrioid, mucinous, clear cell, and mixed categories. The degree of epithelial proliferation and its relation to the stromal component of the tumour are the criteria used for the classification as benign, borderline, or malignant, although most of the reported ovarian cystadenofibromas were benign [1, 4].

Ovarian malignancy is strongly suspected when a patient presents with an ovarian cystic mass with solid components. Ovarian cystadenofibromas are notable from an imaging standpoint because they often have solid components and thus mimic malignant neoplasms. Some investigators have reported that this type of tumour can be diagnosed as malignant based on imaging alone [3–6]. Moreover, these tumours often have the gross appearance of a malignant tumour at the time of surgery. A frozen-section diagnosis may be helpful in many of these cases because a correct diagnosis of cystadenofibroma in the operating room can save the patient from unnecessary extensive surgery. Therefore, recognition of this tumour by clinicians is of utmost importance.

When imaged by ultrasonography or CT, ovarian cystadenofibroma may appear as a complex solid and cystic mass and be difficult to differentiate from malignancy. Thus, MRI may be an essential modality for diagnosing this tumour, especially when the characteristic “black sponge” appearance is observed on T2-weighted images. Outwater et al. reported that the dense fibrous tissue of the solid component of cystadenofibroma showed very low signal intensity on T2-weighted images, similar to skeletal muscle [5, 6]. Cystadenocarcinofibroma had a solid portion with strong enhancement and higher signal intensity compared to benign cystadenofibromas. Signal intensities of the solid portions were higher on the T2-weighted images of malignant tumours than on those of their benign counterparts. In general, the likelihood of malignancy increases with an increasing solid tissue portion and thicker septa and contrast enhancement usually facilitates differentiation between benign and malignant lesions [7, 8].

Given the rarity of ovarian cystadenofibroma, its occurrence may be difficult to detect. Therefore, ovarian cystadenofibroma is one of many tumours subject to differential diagnosis in which preoperative diagnosis by MRI is useful. In addition, frozen sections may be helpful in the intraoperative assessment of ovarian masses to provide guidance for appropriate surgical management.
